# CsMYB67 participates in the flavonoid biosynthesis of summer tea leaves

**DOI:** 10.1093/hr/uhad231

**Published:** 2023-11-17

**Authors:** Ying Ye, Ru-Yi Liu, Xin Li, Xin-Qiang Zheng, Jian-Liang Lu, Yue-Rong Liang, Chao-Ling Wei, Yong-Quan Xu, Jian-Hui Ye

**Affiliations:** Tea Research Institute, Zhejiang University, 866 Yuhangtang Road, Hangzhou 310058, China; Tea Research Institute, Zhejiang University, 866 Yuhangtang Road, Hangzhou 310058, China; Key Laboratory of Tea Biology and Resources Utilization, Ministry of Agriculture, Tea Research Institute Chinese Academy of Agricultural Sciences, Hangzhou 310000, China; Tea Research Institute, Zhejiang University, 866 Yuhangtang Road, Hangzhou 310058, China; Tea Research Institute, Zhejiang University, 866 Yuhangtang Road, Hangzhou 310058, China; Tea Research Institute, Zhejiang University, 866 Yuhangtang Road, Hangzhou 310058, China; State Key Laboratory of Tea Plant Biology and Utilization, Anhui Agricultural University, 130 Changjiang West Road, Hefei 230036, China; Key Laboratory of Tea Biology and Resources Utilization, Ministry of Agriculture, Tea Research Institute Chinese Academy of Agricultural Sciences, Hangzhou 310000, China; Tea Research Institute, Zhejiang University, 866 Yuhangtang Road, Hangzhou 310058, China

## Abstract

Flavonoids are important compounds in tea leaves imparting bitter and astringent taste, which also play key roles in tea plants responding to environmental stress. Our previous study showed that the expression level of *CsMYB67* was positively correlated with the accumulation of flavonoids in tea leaves as exposed to sunlight. Here, we newly reported the function of CsMYB67 in regulating flavonoid biosynthesis in tea leaves. CsMYB67 was localized in the nucleus and responded to temperature. The results of transient expression assays showed the co-transformation of *CsMYB67* and *CsTTG1* promoted the transcription of *CsANS* promoter in the tobacco system. CsTTG1 was bound to the promoter of *CsANS* based on the results of yeast one-hybrid (Y1H) and transient expression assays, while CsMYB67 enhanced the transcription of *CsANS* through protein interaction with CsTTG1 according to the results of yeast two-hybrid (Y2H) and bimolecular fluorescence complementation (BiFC). Thus, CsMYB67-CsTTG1 module enhanced the anthocyanin biosynthesis through up-regulating the transcription of *CsANS*. Besides, CsMYB67 also enhanced the transcription of *CsFLS* and *CsUFGT* through forming transcription factor complexes. The function of *CsMYB67* on flavonoid biosynthesis in tea leaves was validated by gene suppression assay. As *CsMYB67* was suppressed, the transcriptional level of *CsFLS* was greatly reduced, leading to a significant increase in the contents of total catechins and total anthocyanidins. Hence, CsMYB67 plays an important role in regulating the downstream pathway of flavonoid biosynthesis in summer tea leaves.

## Introduction

Tea as a worldwide beverage is produced from the leaves of tea plant *Camellia sinensis* (L.) O. Kuntze. Flavonoids are the main bitter and astringent compounds in teas [1], which also importantly contribute to the various bioactivities of tea; for instance, anti-inflammatory [2], anti-diabetic [3], and cardiovascular protective effects [4]. Flavanols, flavonols, and anthocyanins are the three major subclasses of flavonoid compounds in tea leaves [5], with the general chemical structure C6–C3–C6 of a 15-carbon skeleton [6]. The flavonoid composition of tea varies with tea cultivar [7, 8] and leaf maturity [9], which is also affected by cultivation environmental conditions [10, 11], plucking season [12], as well as tea processing techniques [13, 14].

In fresh tea leaves, catechins are the most abundant flavanols, mainly including epicatechin (EC), epicatechin gallate (ECg), epigallocatechin (EGC), epigallocatechin gallate (EGCg), catechin (C), and catechin gallate (Cg). Second to the contents of catechins, flavonol compounds in tea leaves mainly exist in the form of the *O*-glycosides of quercetin, kaempferol, and myricetin, subsequently followed by anthocyanins (e. g. the glycosyl derivatives of pelargonidin, delphinidin, and cyanidin). Despite the relatively lower contents, flavonol glycosides have extremely low threshold values of astringency as well as great enhancing effect on the bitterness of caffeine [15]. The compounds of these three flavonoid subclasses are generated from the flavonoid biosynthetic pathway consisting of phenylpropanoid and flavonoid biosynthesis pathways. Following the same upstream enzymes in the flavonoid biosynthesis pathway, the downstream branches mediate the biosynthesis of catechins, flavonol glycosides, and anthocyanins [9]. R2R3-MYB, bHLH, WD40, as well as MYB–bHLH–WD40 (MBW) complex are the important transcription factor (TF) regulators of flavonoid biosynthesis [16, 17].

Light and temperature are the key environmental factors to flavonoid biosynthesis in tea plant. Flavonoid compounds are abundantly accumulated in summer tea leaves. Strong illumination promotes the transcriptions of structural genes for flavonoid biosynthesis in tea leaves, such as phenylalanine ammonia-lyase (*CsPAL*), chalcone synthase (*CsCHS*), flavanone 3-hydroxylase (*CsF3H*), flavonoid 3′-hydroxylase (*CsF3′H*), anthocyanidin reductase (*CsANR*), dihydroflavonol reductase (*CsDFR*), flavonol synthase (*CsFLS*), and UDP glucose-flavonoid-3-*O*-glycosyltransferases (*CsUFGT*) [10, 18–20]. It has been reported that CsMYB4, CsMYB12, and CsMYB7 regulated the flavonoid biosynthesis in tea plants [18, 20], among which the transcription of CsMYB12 was activated by UV B through stabilizing CsHY5. In addition to strong illumination in summer, high temperature also enhances the biosynthesis of flavonoids in tea leaves [21]. The biosynthesis of galloylated catechins is promoted by high temperature resulting in the elevated ratio of tea polyphenols to total amino acids, which is adverse to tea quality [22]. Up to the time of writing, many studies have investigated the impact of a sole environmental factor on flavonoid biosynthesis in tea plants under controlled conditions, through supplementing/deleting certain light spectrums [18], or adjusting temperature treatment [21]. However, in the field crop production, multiple environmental factors are usually involved due to changes of weather and climate. Strong illumination in summer is accompanied by high temperature. Thus, in production practice, the photothermal-enhanced biosynthesis of flavonoids in tea leaves may not be fully explained by the regulatory mechanisms of flavonoid biosynthesis due to either light or temperature change in a climatic chamber. The molecular mechanisms underlying the greatly accumulated flavonoids in summer tea leaves are still unclear, and the crucial TFs responsible for promoting flavonoid biosynthesis need investigation.

Our previous study found that under different field shade treatments without leaf temperature control, five TFs were screened out of 40 flavonoid biosynthesis-related TFs according to their multiple significant correlations with the levels of various catechins and flavonol glycosides, namely *CsMYB12* (TEA009412.1), *CsMYB67* (TEA015433.1), *CsC1* (TEA004608.1), *CsMYB4* (TEA033191.1), and *CsKTN80.4* (TEA033903.1) [10]. Considering the high transcriptional expressions in the naturally grown tea leaves (control) [10], these five photothermal-inducible TFs may be involved in the flavonoid biosynthesis of summer tea leaves and deserve further verification. In the present study, we investigated the interactions between these five TF candidates and the structural genes for flavonoid biosynthesis using yeast one-hybrid assays (Y1H) and transient expression assays, and found a new TF *CsMYB67* regulating flavonoid biosynthesis. Furthermore, CsMYB67 was characterized and the function was investigated. Biochemical analysis revealed that CsMYB67 promoted the transcription of *CsANS* through forming complex with CsTTG1 based on the results of yeast two-hybrid assays (Y2H) and bimolecular fluorescence complementation (BiFC). The function of *CsMYB67* on flavonoid biosynthesis in tea leaves was also testified by gene suppression assay.

## Results

### Identification of the key genes correlated with flavonoids

**Figure 1:**
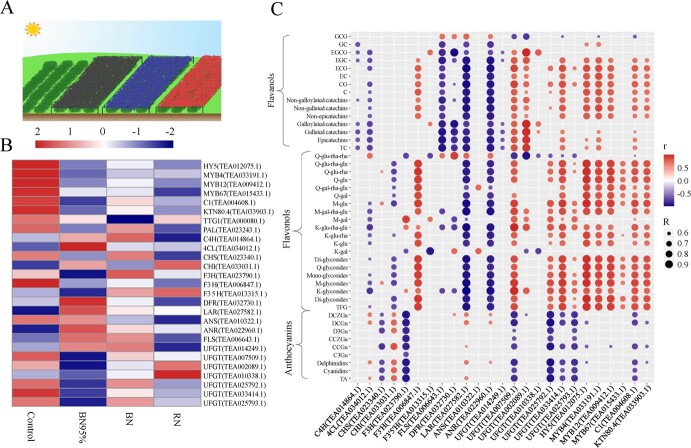
The effect of different shade treatments on the flavonoid biosynthesis in tea leaves. **A** The sketch map of different shade treatments. **B** The heatmap of flavonoid biosynthesis-related gene expression levels. **C** The correlations between flavonoid contents and relevant gene expression levels. Pearson’s correlation was analysed using R scripts. r: correlation coefficient; R: absolute value of correlation coefficient; GCG: gallocatechin gallate; TC: total catechins. M-gal-rha-glu: myricetin-3-*O*-glucosyl-rhamnosyl-galactoside; M-gal: myricetin-3-*O*-galactoside; M-glu: myricetin-3-*O*-glucoside; K-glu-rha-glu: kaempferol-3-*O*-glucosyl-rhamnosyl-glucoside; K-gal: kaempferol-3-*O*-galactoside; K-glu-rha: kaempferol-3-*O*-rhamnosyl-glucoside; K-glu: kaempferol-3-*O*-glucoside; Q-gal-rha-glu: quercetin-3-*O*-glucosyl-rhamnosyl-galactoside; Q-glu-rha: quercetin-3-*O*-rhamnosyl-glucoside; Q-glu-rha-rha: quercetin-3-*O*-rhamnosyl-rhamnosyl-glucoside; Q-glu-rha-glu: quercetin-3-*O*-glucosyl-rhamnosyl-glucoside; Q-gal: quercetin-3-*O*-galactoside; Q-glu: quercetin-3-*O*-glucoside; TFG: total flavonol glycosides.

Tea plants were treated by black net, blue net, and red net in the field (Fig. 1A), the samples of which were termed BN95%, BN, and RN respectively, using the tea plants without shade treatment as control. The contents of catechins and flavonol glycosides in different tea samples are achieved from our previous work [10]. The control sample had the highest levels of total catechins (TC) and total flavonol glycosides (TFG), while BN95% contained the lowest contents of TC and TFG. The contents of anthocyanins were also analysed and shown in Table 1. BN95% contained the most abundant total anthocyanins (TA) at the content of 514.38 ± 23.22 μg/g DW (dry weight), followed by RN (439.11 ± 27.74 μg/g DW) and control (350.41 ± 12.80 μg/g DW), while BN contained the lowest content of TA (294.60 ± 2.93 μg/g DW, Table 1). The corresponding transcriptome data were achieved from the BIG data center [10], and were pictured in a heatmap (Fig. 1B). Obviously, the expression levels of *CsHY5* (TEA012075.1), *CsMYB4* (TEA033191.1), *CsMYB12* (TEA009412.1), *CsMYB67* (TEA015433.1), *CsC1* (TEA004608.1), *CsKTN80.4* (TEA033903.1), *CsF3’H* (TEA006847.1), *CsANS* (TEA010322.1), and *CsUFGT* (TEA007509.1, TEA033414.1) in the control were much higher than those in the shade-treated samples, suggesting that the transcription of these flavonoid biosynthesis-related genes were elevated due to the photothermal effect of sun exposure. Fig. 1C shows the correlations between genes and metabolites in flavonoid biosynthesis. The transcript abundances of *CsF3H* (TEA023790.1) and *CsUFGT* (TEA007509.1, TEA033414.1) were positively correlated with the levels of most catechins and flavonol glycosides, while *CsFLS* (TEA006643.1) had negative correlations with catechin contents. For TFs, the major catechin and flavonol glycoside contents were positively related with the expression levels of *CsHY5*, *CsMYB4*, *CsMYB12*, *CsC1*, and *CsKTN80.4*, while relatively fewer flavonoid compounds were correlated with the transcript abundance of *CsMYB67*. Nevertheless, considering the multiple correlations with various flavonoid compounds, these five TFs were then selected for further investigations on their roles in flavonoid biosynthesis.

**Table 1:** The anthocyanin contents of different tea samples.

Anthocyanins (μg/g DW)	Control	BN95%	BN	RN
D3Ga	21.27 ± 0.64b	27.13 ± 1.74a	15.64 ± 1.38c	26.22 ± 1.07a
C3Ga	23.06 ± 1.02a	23.89 ± 1.24a	17.03 ± 2.13b	22.06 ± 1.63a
DCZGa	41.98 ± 1.47c	68.57 ± 3.27a	33.79 ± 0.85d	50.77 ± 3.31b
CCZGa	24.05 ± 1.09b	29.20 ± 0.34a	18.96 ± 1.28c	24.73 ± 0.73b
DCGa	148.68 ± 4.51c	245.32 ± 10.96a	127.63 ± 2.56c	201.73 ± 15.59b
CCGa	91.38 ± 4.74b	120.27 ± 6.23a	81.55 ± 1.12b	113.61 ± 6.07a
TA	350.41 ± 12.80c	514.38 ± 23.22a	294.60 ± 2.93d	439.11 ± 27.74b

Different alphabetic letters (a, b, c, d) in the same row indicate significant difference (*P* < 0.05). BN95%: black net 95% shade-treated sample; BN: blue net shade-treated sample; RN: red net shade-treated sample; DW: dry weight; D3Ga: delphinidin-3-*O*-β-D-galactopyranoside; C3Ga: cyanidin-3-*O*-β-D-galatopyranoside; DCZGa: delphinidin-3-*O*-β-D-(6-(*Z*)-*p*-coumaroyl)galactopyranoside; CCZGa: cyanidin-3-*O*-β-D-(6-(*Z*)-*p*-coumaroyl)galactopyranoside; DCGa: delphinidin-3-*O*-β-D-(6-(*E*)-*p*-coumaroyl)galactopyranoside; CCGa: cyanidin-3-*O*-β-D-(6-(*E*)-*p*-coumaroyl)galactopyranoside; TA: total anthocyanins.

### Characterization of the activating role of TF candidates in flavonoid biosynthesis

**Figure 2:**
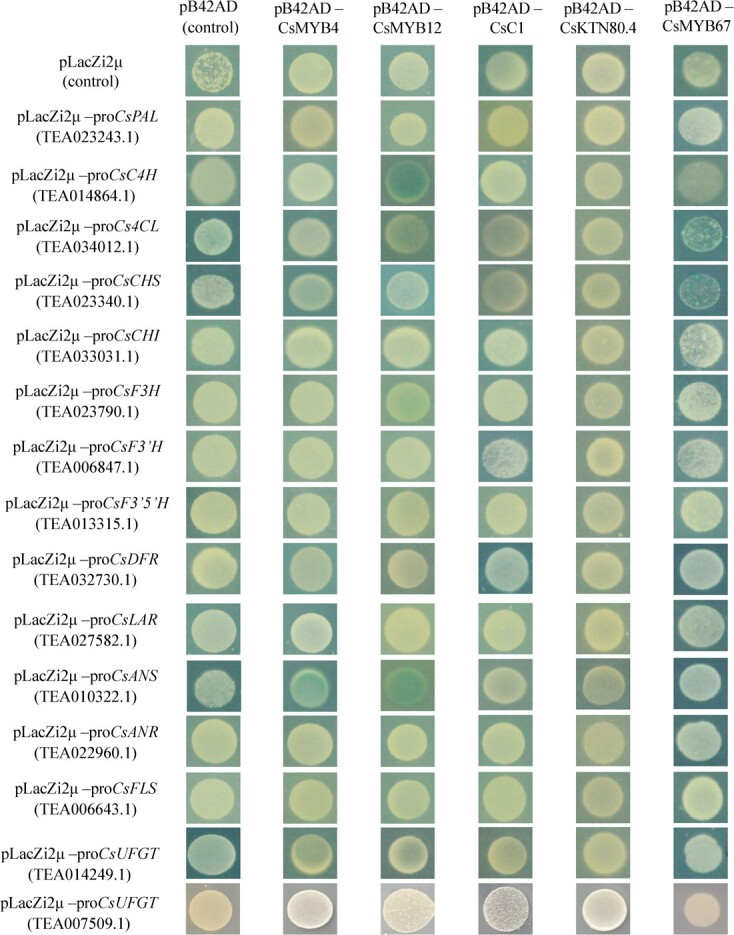
The Y1H results of the interactions between candidate transcription factors and the promoters of structural genes for flavonoid biosynthesis. pLacZi2μ and pB42AD were used as negative controls. SD/Gal/Raf/-Ura/−Trp/+X-gal selective media indicates yeast nitrogen base containing galactose, raffinose, and X-gal, without uracil (Ura) and tryptophan (Trp).

**Figure 3:**
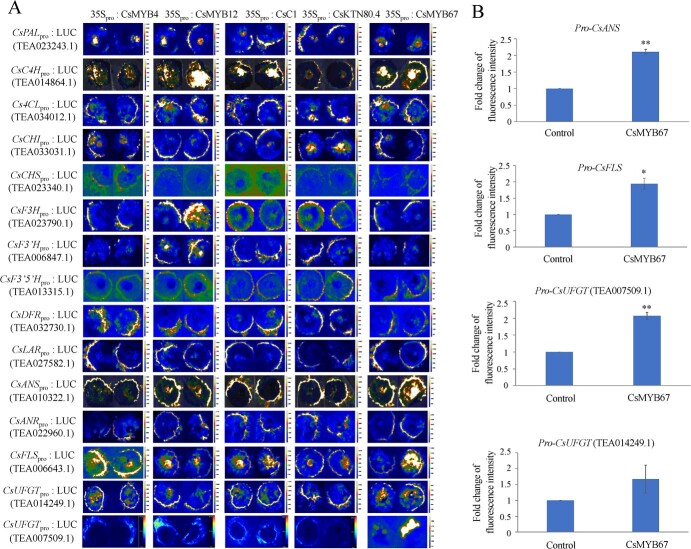
The transient expression results of the interactions between CsMYB67 and the promoters of structural genes for flavonoid biosynthesis. **A** Transient expression assay. Left: an empty vector as a negative control; Right: co-infusion with an effector and a pGreenII-0800-LUC-pro as a reporter. **B** Fold change of the fluorescence intensity for the promoters of *CsANS*, *CsFLS*, and *CsUFGT* (TEA007509.1, TEA014.249.1). ^*^P < 0.05, ^**^P < 0.01.

Fig. 2 shows the Y1H result of five TF candidates and structural genes. Only CsMYB12 was bond to the promoters of *CsC4H* (TEA014864.1), *CsF3H* and *CsANS* based on the Y1H result. The activating effects of these five TF candidates were further verified by transient expression in the tobacco systems (Fig. 3A). The co-infusion of *CsMYB12* significantly elevated the fluorescence intensities for the promoters of *CsC4H* and *CsF3H*, while co-infusion of *CsMYB4*, *CsC1*, and *CsKTN80.4* exerted no obvious influence on the fluorescence intensities for all the tested structural genes. This result is generally consistent with the Y1H result in Fig. 2. Despite no direct interaction occurring between CsMYB67 and the promoter of *CsANS* in the yeast system (Fig. 2), the transient expression of CsMYB67 could dramatically increase the fluorescence intensities for the promoters of *CsANS* (TEA010322.1), *CsFLS* (TEA006643.1), and *CsUFGT* (TEA007509.1) in the tobacco leaves, implying that CsMYB67 activated the transcription of the promoters of *CsANS* (TEA010322.1), *CsFLS* (TEA006643.1), and *CsUFGT* (TEA007509.1) (Fig. 3A). The fluorescence intensities for the promoters of *CsANS* (TEA010322.1), *CsFLS* (TEA006643.1), and *CsUFGT* (TEA007509.1) were significantly elevated by 2.11-fold, 1.94-fold, and 2.07-fold due to the co-transformation of CsMYB67, compared with empty vectors (Fig. 3B). The results of transient expression and Y1H assays showed that CsMYB67 might indirectly interact with the promoters of *CsANS* (TEA010322.1)*, CsFLS* (TEA006643.1), and *CsUFGT* (TEA007509.1) through forming TF complexes. The CsMYB12-mediated flavonoid biosynthesis has been reported in tea plants [18, 23]; however, the role of CsMYB67 has not been verified in tea plant. Hence, CsMYB67 was selected for further study.

### Molecular characterization, subcellular localization, and induction of *CsMYB67*

**Figure 4:**
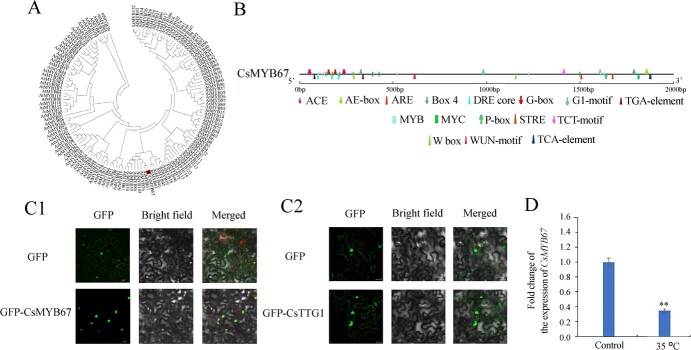
**.** The information of CsMYB67 and CsTTG1. **A** Phylogenetic analysis. **B** The *cis*-acting elements in the promoters of CsMYB67. **C1**, **C2** Subcellular localization of CsMYB67 and CsTTG1 in tobacco leaf cells. Scale bar = 20 μm. **D** The effect of temperature on the expression of CsMYB67 in tea leaves. ^**^P < 0.01.

The result of ProtParam software analysis indicated that the coding sequence of *CsMYB67* cloned from the tea leaves of cv. *Fudingdabaicha* encodes a protein comprising 333 amino acid residues, with a molecular weight of 37.50 kD and an isoelectric point of 6.53 as predicted. The phylogenetic analysis indicated that CsMYB67 was homologous to AtMYB67 (Fig. 4A), which was clustered with AtMYB67, AtMYB26, and AtMYB103. The function of AtMYB67 in regulation of flavonoids has not been reported, whereas AtMYB26 is likely to be involved in regulating phenylpropanoid biosynthesis [24]. The conserved R2 and R3 domains were found in the amino acid sequence of CsMYB67 (Fig. S1, see online supplementary material), where the R2 region was located at amino acid residues 13–60 and R3 was located at amino-acid residues 66–109. Fig. 4B showed the predicted binding sites for MYB, MYC, and WRKY, as well as *cis*-acting elements located in the promoter of *CsMYB67*, such as light-responsive elements (ACE, G-box, AE-box, GT1-motif, Box 4, and TCT-motif), stress response element STRE, dehydration-responsive element DRE core, as well as hormone response elements (P-box, TCA-element, and TGA-element). In a word, there are 10 light-responsive elements, five hormone response elements, and four temperature-responsive elements present in the promoters of *CsMYB67*, indicating that the transcription of *CsMYB67* could be affected by various factors, like light, temperature, and phytohormones. Fig. 4 C1 reveals that CsMYB67 was localized in the nucleus.

HY5 as an important light signal transduction element is crucial to flavonoid biosynthesis in plants [25]. Fig. S2A and B (see online supplementary material) showed no interaction occurred between CsMYB67 and CsHY5 based on the results of Y2H and BiFC assays. Moreover, CsHY5 was not bound to the promoter of CsMYB67 (Fig. S2C, see online supplementary material). Hence, the light-responsible CsHY5 may not regulate the transcription of *CsMYB67* directly, and then we investigated the effect of temperature on the induction of *CsMYB67* (Fig. 4D). In the environmental chamber study, *CsMYB67* showed sensitivity to temperature, which was transcriptionally suppressed at high temperature (35°C), compared with control.

### CsMYB67 formed complex with CsTTG1 to promote *CsANS* transcription in tobacco leaves

**Figure 5:**
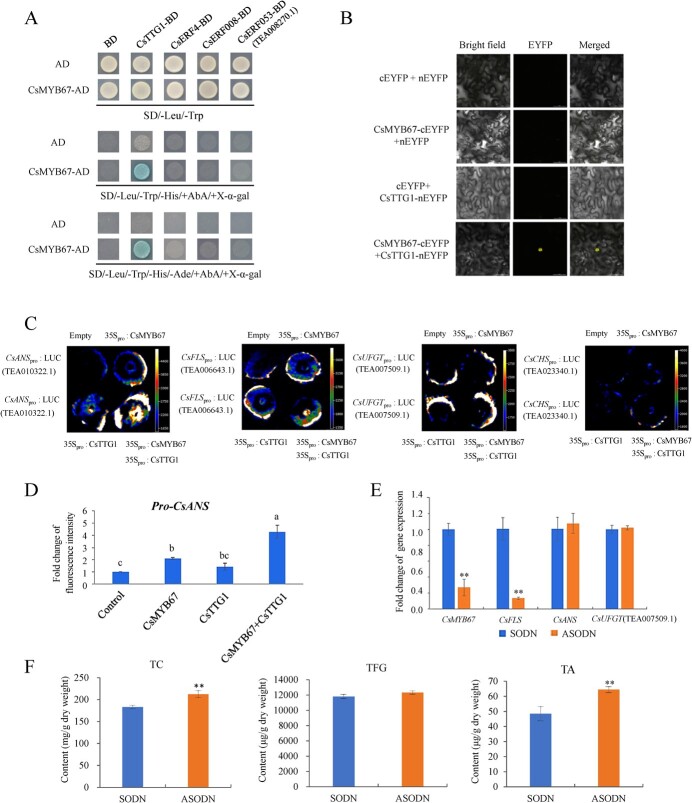
CsMYB67-CsTTG1 interaction and its impact on the transcription of CsANS. **A** The Y2H result for CsMYB67 and CsTTG1. **B** The BiFC result for CsMYB86 and CsTTG1. Scale bar = 50 μm. **C, D** Transient expression assay. **E** Expression of CsMYB67 and genes in the control (sODN) and CsMYB67/AsODN tea leaves. **F** The contents of TC, TFG, and TA in the control (sODN) and CsMYB67/AsODN tea leaves. TC: total catechins; TFG: total flavonol glycosides; TA: total anthocyanins. Different alphabetic letters (a, b, c) indicated significant difference (*P* < 0.05). ^**^P<0.01.

The above results showed that the transcription of *CsANS*, *CsFLS*, and *CsUFGT* (TEA007509.1) might be regulated by CsMYB67 complex. In order to find the TF component, we searched relevant literature and found that TTG1 and ERFs participated in the co-regulation of anthocyanin biosynthesis with MYBs in plants, such as eggplant [26] and pear fruits [27]. Through sequence alignment in TPIA, we identified and cloned the coding sequence of *CsTTG1* (TEA000080.1), *CsERF4*, *CsERF008*, and *CsERF053* (TEA008270.1) in tea plant. The Y2H result showed that only the CsMYB67 + CsTTG1 group had blue positive colonies appearing on both selective media, suggesting that an interaction occurred between CsMYB67 and CsTTG1 (Fig. 5A). This result was validated by BiFC assay (Fig. 5B). In the tobacco system, CsMYB67 and CsTTG1 activated the transcription of *CsANS* promoter, respectively, and the activating effect was greatly enhanced due to the co-transformation (Fig. 5C). However, no obvious enhancing effect of CsMYB67 and CsTTG1 co-transformation was observed for *CsCHS* (TEA023340.1), *CsFLS*, and *CsUFGT* (TEA007509.1) (Fig. 5C). For *CsANS*, the calculated fluorescence intensity of CsMYB67 + CsTTG1 combination group was significantly elevated, compared with transformation with either CsMYB67 or CsTTG1, while control had the lowest fluorescence intensity (Fig. 5D). Hence, the transcription of *CsANS* was directly mediated by CsMYB67-CsTTG1 module, while *CsFLS* and *CsUFGT* (TEA007509.1) were indirectly regulated by CsMYB67.

The phylogenetic analysis showed CsTTG1 has high similarity to VcTTG1 and AcTTG1 [28]. VcTTG1 and AcTTG1 are, respectively, related with the anthocyanin biosynthesis in blueberry [29], and *Aglaonema commutatum* [30]. Fig. S1B (see online supplementary material) indicated that CsTTG1 belongs to the WD40 family. CsTTG1 was localized in both nucleus and cytoplasm (Fig. 4C2). CsTTG1 only interacted with the promoters of *CsCHS* and *CsANS* based on the Y1H result (Fig. S3, see online supplementary material), which were further testified by transient expression assay (Fig. 5C). The transient expression assay validated the activating effect of CsTTG1 on the transcription of *CsANS* promoter rather than the *CsCHS* promoter.

### Characterization of *in vivo* function of suppressed *CsMYB67* in flavonoid biosynthesis

The antisense oligodeoxynucleotide (AsODN) suppression assay was used to suppress the expression of *CsMYB67* in tea leaves. Fig. 5E indicated that the expression level of *CsMYB67* in the *CsMYB67/AsODN* tea leaves was inhibited by 73.2% compared with control (sODN), suggesting that the transcription of *CsMYB67* in tea leaves was dramatically inhibited through AsODN suppression. In contrast to the sODN group, the expression level of *CsFLS* in the *CsMYB67/AsODN* tea leaves was greatly reduced by 86.5%, without an obvious impact on the transcript abundances of *CsANS* and *CsUFGT* (TEA007509.1) (Fig. 5E). This suggests that the transcriptions of *CsANS* and *CsUFGT* (TEA007509.1) were barely affected as *CsMYB67* was suppressed. Fig. 5F shows the contents of TC, TFG, and TA in the control (sODN) and CsMYB67/AsODN tea leaves, and the contents of individual flavonoids are listed in Table S1 (see online supplementary material). The CsMYB67/AsODN tea leaves contained 212.45 mg/g DW and 64.48 μg/g DW of TC and TA, which were significantly higher than 182.99 mg/g DW and 48.43 μg/g DW of control. The increase of TC in the CsMYB67/AsODN tea leaves was mainly attributed to the increase of EGCG, being 96.01 mg/g DW compared with 77.66 mg/g DW of control. Similarly, the increase of TA in the CsMYB67/AsODN tea leaves was mainly due to the elevated content of delphinidin-3-*O*-β-D-(6-(*E*)-*p*-coumaroyl) galactopyranoside (DCGa). By contrast, no significant difference in the TFG content was observed between CsMYB67/AsODN tea leaves and control (Fig. 5F). Specifically, the contents of myricetin-3-*O*-glucosyl-rhamnosyl-galactoside (M-gal-rha-glu) and myricetin-3-*O*-glucoside (M-glu) were reduced due to the suppression of CsMYB67, whereas the contents of quercetin-3-*O*-glucosyl-rhamnosyl-galactoside (Q-gal-rha-glu) and kaempferol-3-*O*-glucosyl-rhamnosyl-glucoside (K-glu-rha-glu) were significantly increased (Table S1, see online supplementary material). Thus, the *CsMYB67* suppression-induced trade-offs among certain flavonol glycosides in tea leaves led to the almost unchanged content of TFG.

## Discussion

### Shade treatment attenuated flavonoid biosynthesis in tea leaves

Shading is effective to cut down illumination and reduce the leaf temperature of tea plants. In our study, the highest contents of TC and TFG were achieved in the control sample, while their lowest contents were achieved in BN95% [10]. Thus, shade treatment attenuated the accumulation of major flavonoids in tea leaves, which was consistent with the generally down-regulated expressions of flavonoid biosynthesis-related genes, including *CsPAL*, *CsCHS*, *CsF3H*, and *CsF3′H* (Fig. 1B). Wang *et al.* [19] also reported that shading greatly down-regulated the transcription of *CsPAL*, *CsCHS*, *CsF3H*, *CsF3′H*, *CsANR1*, and *CsUFGT* in tea leaves. In other words, strong illumination promoted flavonoid biosynthesis in tea leaves [19, 31]. In tea plants cv. Shuchazao, CsMYB12 was bound to the promoters of *CsFLS* and *CsUFGT* and activated their transcriptions [18]. In our study, CsMYB12 activated the transcriptions of *CsC4H* and *CsF3H* through binding to their promoters. The difference could be attributed to different tea cultivars used. This is also a plausible explanation for the differential responses of different tea cultivars to shade treatment, in terms of flavonoid biosynthesis [8].

### The putative regulation mechanism of CsMYB67

As at the date of this writing, MYB67 is newly reported in tea plants. MYB67 was associated with suberin biosynthesis in different plants, such as poplar [32] and apple [33]. The biosynthesis of suberin monomers partially involves phenylpropanoid pathways [34], and phenylpropanoid pathway is the upstream route for flavonoid biosynthesis. Our study validated the role of CsMYB67 in regulating flavonoid biosynthesis of tea leaves through field experiment and gene suppression assay. *CsMYB67* was transcriptionally up-regulated due to the photothermal effect in the field experiment of tea plants (Fig. 1B). However, in the environmental chamber study, the expression of *CsMYB67* in the one-year-old tea plants was suppressed at the high temperature (Fig. 4D). This suggests that the transcription of *CsMYB67* is responsive to temperature. Diverse *cis*-activating elements were present in the promoter region of *CsMYB67*, like stress response element STRE and dehydration-responsive element DRE core, suggesting that the transcription of *CsMYB67* could be activated by environmental stresses (e.g. drought). MYB67 has been related to the biosynthesis of suberin that prevents water loss from plant tissues and provides protection against various threats. Thus, MYB67 has the potential contribution to stress resistance of plants. In our study, the different performance of *CsMYB67* in the field experiment and the environmental chamber study could be attributed to the different stress resistances between three-year-old field-grown tea plants and one-year-old tea seedlings. Apparently, the three-year-old tea plants have higher resistance to high temperature-induced drought, with the elevated transcriptional level of stress resistance-related *CsMYB67*, compared to the vulnerable one-year-old tea seedlings cultivated in the environmental chamber. Nevertheless, the function of *CsMYB67* in the drought resistance of tea plants still needs further investigations, in terms of its regulatory effects on the biosynthesis of suberin and antioxidants like flavonoid compounds. For flavonoid biosynthetic pathway, the infusion of CsMYB67 in the tobacco leaves only activated the transcription of the promoters of *CsANS*, *CsFLS*, and *CsUFGT*, respectively, without direct interaction between CsMYB67 and the promoters. *CsFLS* and *CsUFGT* are the crucial structural genes for the biosynthesis of flavonol glycosides [18], while *CsANS* participates in the late biosynthesis of anthocyanins [1]. Thus, CsMYB67 importantly participates in the downstream pathway of flavonoid biosynthesis through forming TF complex.

### CsMYB67 enhanced the anthocyanin biosynthesis by forming complex with CsTTG1

TTG1 as a WD40 repeat protein is involved in many physiological processes of plants, such as secondary metabolisms and responses to biotic and abiotic stresses [35, 36]. TTG1 may interact with R2R3-MYB and bHLH to regulate downstream target genes of flavonoid biosynthesis [37, 38]. VfTTG1 mediated the tannin and anthocyanin biosynthesis in faba bean [35]. Typical purple leaf tea cultivars cv. *Zijuan* and cv. *Ziyan* had higher transcript levels of *CsANS, CsUGT, CsTTG1*, *CsPAP1*, and *CsTT8* than regular green leaf cultivar cv. *Yunkang* based on transcriptome [39]. The formation of CsMYB75 or CsMYB86-CsTT8/CsGL3-CsTTG1 MBW complexes elevated the expression level of *CsDFR* in tea plant, promoting the biosynthesis of anthocyanins and catechins [40]. High temperature-inducible *CsJAZ6* directly interacted with CsEGL3 and CsTTG1, leading to the reduction of catechin contents in the one-year-old tea plants [41]. In the present study, the transcription of *CsANS* was coordinately changed with the transcriptions of *CsMYB67* and *CsTTG1*, reaching the expression peak in the control sample (Fig. 1B). However, as the expression of *CsMYB67* was suppressed, the transcription of *CsANS* in tea leaves was hardly affected. This was possibly due to the activating effect of solo CsTTG1 on the transcription of *CsANS*. Thus, highly expressed *CsMYB67* can promote the transcription of *CsANS* through forming MBW complex with CsTTG1, while *CsMYB67* at the low expression level hardly affects the transcription of *CsANS*. Temperature-sensitive *CsMYB67* interacts with CsTTG1 for fine-turning the biosynthesis of anthocyanins in tea leaves.

### The photothermal effect on the distribution of catechins, flavonol glycosides, and anthocyanins in tea leaves

**Figure 6:**
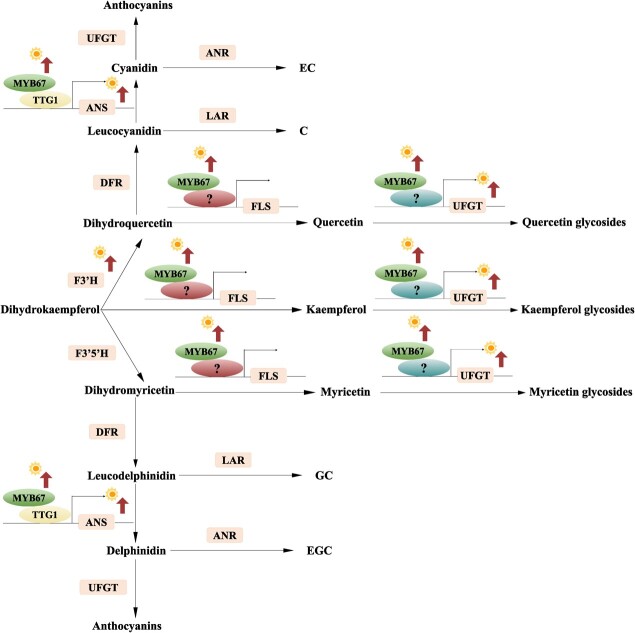
The role of CsMYB67 in flavonoid biosynthesis of tea leaves.

Despite the generally attenuated flavonoid biosynthesis in tea plants under shade treatment, different flavonoid subclasses may have differential behaviors. In Fig. 1C, the expression of *CsMYB67* is positively related with the contents of various flavonol glycosides, implying that the highly expressed *CsMYB67* exposed to sun is likely to promote the biosynthesis of flavonol glycosides. The result of gene suppression study in Fig. 5E showed that the transcription of *CsFLS* in the CsMYB67/AsODN tea leaves was largely mediated by CsMYB67. FLS is the key enzyme converting dihydroflavonols to flavonols, which is an important step in the biosynthetic pathway of flavonol glycosides. The up-regulated transcription of *CsFLS* in tea leaves led to the enhanced biosynthesis of flavonol glycosides, which cost higher amounts of the common precursors divided from the biosynthesis of catechins and anthocyanins. Negative correlations were observed between the expressions of *CsFLS* and the contents of most catechins rather than flavonol glycosides (Fig. 1C), suggesting that the split-flow of common precursors might be an important factor affecting the biosynthesis of catechins while FLS reaction may not be the rate-limiting step for the biosynthesis of flavonol glycosides. Accordingly, the highly expressed *CsUFGTs* in the control sample were positively correlated with the contents of various flavonol glycosides (Fig. 1C). Our previous studies also reported that flavonol glycosides were sensitive to the strong illumination and high temperature in summer [8, 10]. Moreover, as *CsMYB67* was suppressed, the transcriptions of *CsANS* and *CsUFGT* (TEA007509.1) were hardly affected, which was possibly due to the indirect interaction of CsMYB67 with *CsANS* and *CsUFGT*. CsTTG1 directly activated the transcription of *CsANS* without the presence of CsMYB67*,* while the component of MBW complex for regulating *CsUFGT* is still unclear. Fig. 6 depicts the role of CsMYB67 in the flavonoid biosynthesis of tea leaves. As tea plants face strong illumination and high temperature in summer, CsMYB67 not only activates the transcriptions of *CsFLS* and *CsUFGT* resulting in the greatly enhanced biosynthesis of flavonol glycosides, but also fine-tune the biosynthesis of anthocyanins, in addition to its potential role in stress resistance. The high sensitivity of flavonol glycosides responding to external environment stresses, like strong light and high temperature, has been reported in plants, e.g., Kale [42], the cotyledons of Buckwheat seedlings [43], and apple fruit [44]. For anthocyanins, the environmental chamber experiment indicated that high temperature down-regulated the expression of *CsMYB67* that activated the transcription of *CsANS*, which meant high temperature is adverse to anthocyanin biosynthesis. Due to shading, BN95% produced from the tea leaves with relatively lower leaf temperature contained more anthocyanins than the control sample (Table 1). It has been reported that high temperature suppressed the biosynthesis of anthocyanins [41, 45, 46], while low temperature is propitious to anthocyanin biosynthesis in various plants, e.g., red-skinned grape [47] and purple head Chinese Cabbage [48]. The CsMYB67-mediated distribution of catechins, flavonol glycosides, and anthocyanins from the common precursors in tea leaves was also validated by the gene suppression study (Table S1, see online supplementary material). At the metabolic level, the suppression on *CsFLS* transcription in the CsMYB67/AsODN tea leaves led to the increased contents of TC and TA, as more common precursors might be diverted to the biosynthesis of catechins and anthocyanins rather than flavonol glycosides. This deduction is testified by the phenomenon that the contents of EGCG and DCGa from the same precursor of dihydromyricetin were significantly increased, whereas the contents of myricetin glycosides greatly declined (Table S1, see online supplementary material). From this, *CsFLS* also mediates the composition of flavonoid subclasses through reverse redistributing the common precursors. The trade-off between increased quercetin/kaempferol glycosides and reduced myricetin glycosides resulted in the relatively stable level of TFG in the CsMYB67/AsODN tea leaves (Fig. 5F). Hence, the biosynthesis of flavonoid subclasses and the trade-offs in between are not only mediated by the key enzymes in the biosynthetic pathway, but also affected by the source of precursors. We are still investigating the unknown TF components that form the complex with CsMYB67 to modulate the transcription of *CsFLS*.

## Materials and methods

### Plant materials and sampling

The tea samples were obtained from our previous study [10]. Briefly, tea plants (cv. *Fudingdabaicha*, 3-year-old), grown at the Songyang tea plantation of Lishui Academy of Agricultural Sciences were shaded for 20 days using different shade nets, including black nets, blue nets and red nets with the shade percentage of 95%. Accordingly, the samples (the second leaf under the apical bud) were termed BN95%, BN, and RN, using the tea plants grown in sunlight as control. All the samples had three biological replicates.

### Analysis of flavonoid compounds in tea leaves

Anthocyanins analysis. The freeze-dried tea samples (80 mg) were ground in liquid nitrogen and then extracted with 0.8 mL of extraction solution (methanol: HCl = 99:1) at 50°C for 30 min. The solution was centrifuged (13 300 rpm, 20 min), and the supernatant was filtrated through a 0.22 μm membrane before submission to an UltiMate™ 3000 HPLC system (Thermo Fisher Scientific Corporation, Lenexa, Kansas, USA). The conditions of HPLC were: Agilent TC-C18 (2) column (5 μm, 4.6 mm × 250 mm), injection volume 10 μL, column temperature 35°C, mobile phase A = 0.5% formic acid+99.5% water (*v/v*), mobile phase B = 40% acetonitrile+60% water (*v/v*). Linear gradient elution was programmed from 68% (*v*) A/32% (*v*) B to 52% (*v*) A/48% (*v*) B during 60 min. An external standard method was used for quantification of the corresponding anthocyanins with the same aglycone (λ = 520 nm), using delphinidin 3-galatoside chloride and cyanidin 3-*O*-galactoside as references.

Catechin and flavonol glycoside analysis. The freeze-dried tea sample (0.15 g) was ground and extracted with 25 mL of 50% (v/v) ethanol solution (100 rpm, 70°С, 30 min). After centrifugation (12 000 rpm, 4°С, 15 min), the supernatants were analysed by ultra-high-performance liquid chromatography–diode array detector–tandem mass spectrometry (UPLC–DAD–MS, Waters Corporation, Milford, MA, USA) according to our previous published method [10].

### Cloning of promoters and TFs

Genomic DNA and RNA were extracted from tea samples. Gene sequences were retrieved from the TPIA (web site: http://tpdb.shengxin.ren/). The coding sequence of TFs were cloned, including *CsHY5* (TEA012075.1), *CsMYB4* (TEA033191.1), *CsMYB12* (TEA009412.1), *CsMYB67* (TEA015433.1), *CsC1* (TEA004608.1), *CsKTN80.4* (TEA033903.1), *CsTTG1* (TEA000080.1). The promoter fragments (approximately 2000 bp) of 20 structural genes for flavonoid biosynthesis were cloned. Table S2 (see online supplementary material) lists the primers for cloning. The fragment of the promoter (∼2000 bp) was amplified from the cv. ‘*Fudingdabaicha*’ genome DNA by PCR. The PCR program was: 98°C for 2 min, 98°C for 10 s, 60°C for 5 s, 68°C for 20 s, 35 cycles, and 68°C for 2 min. The purified PCR product was ligated into the relevant vector and transformed into *Escherichia coli* DH5α competent cells for sequencing.

### Bioinformatic analysis

#### *Cis*-acting element prediction

The *cis*-acting elements in the cloned promoters were predicted by the PlantCARE online site at http://bioinformatics.psb.ugent.be/webtools/plantcare/html/.

#### Prediction of the physicochemical properties of proteins

The ProtParam tool (https://web.expasy.org/protparam/) was employed to predict the physicochemical properties of CsMYB67 and CsTTG1 proteins, including hydrophilicity, molecular mass, and isoelectric point.

#### Amino acid sequence and phylogenetic analysis methods

Amino acid sequences of MYB transcription factors in *Arabidopsis thaliana* were retrieved from the published database of the *Arabidopsis* Information Resource (TAIR) (https://www.arabidopsis.org/). The construction of phylogenetic tree was performed on the Molecular Evolutionary Genetics Analysis (MEGA) 7.0 software (Mega Limited, Auckland, New Zealand), using the neighbor-joining method (1000 bootstrap replicates).

### Subcellular localization

The coding sequences of *CsMYB67* and *CsTTG1* were amplified by PCR and inserted into pCV-GFP-N1 expression vector which contains the green fluorescent protein (GFP) reporter gene. The plasmids were introduced into *Agrobacterium tumefaciens* (GV3101) to select a positive colony for infiltration according to the published method [49]. The construct was transiently expressed in *Nicotiana benthamiana* leaves by the infiltration of *A. tumefaciens* (GV3101). After 44–48 h, the fluorescence signals from tobacco leaf epidermis were examined by a confocal laser scanning electron microscope (Zeiss LSM880).

### Effect of temperature on the expression of *CsMYB67*

Because no direct interaction occurred between CsMYB67 and CsHY5, high temperature treatments were employed to investigate the induction of CsMYB67 expression. One-year-old tea seedlings (cv. Fudingdabacha) were placed in the plastic pots and domesticated in the environmental chamber under the following conditions: 25/20°C air temperature (day/night), 600 μmol m^−2^ s^−1^ photosynthetic photo flux density (PPFD), 12 h/12 h photoperiod (day/night) and 80% relative humidity. Then, the tea seedlings were placed at 35°C air temperature in the environmental chamber for 24 h high temperature treatment, and the second leaves were collected, using the second leaves of tea seedlings constantly grown at 25/20°C as control. Five independent biological replicates were collected.

### Y1H assay

The coding sequences of *CsMYB4*, *CsMYB12*, *CsMYB67*, *CsC1*, *CsKTN80.4,* and *CsTTG1* were inserted into the pB42AD vectors, respectively, and the promoter fragments of flavonoid biosynthetic genes were constructed into the pLacZi2μ vectors. The Y1H assay was conducted according to the reported method [1]. In brief, the constructed vectors (pLacZi2μ and pB42AD vectors) were co-transformed into the yeast strain EGY48 competent cells, and the empty vectors were co-transformed as the negative control. Then, the yeast cells were cultured on the SD/-Ura/−Trp media (lack of Ura and Trp). After culture at 29°C for 72 hours, the positive colonies were selected and further cultured on the selective SD/Gal/Raf/−Ura/−Trp/+X-gal media plates containing 0.11 M galactose (Gal), 0.02 M raffinose (Raf), 10 × buffered salt (0.26 M Na_2_HPO_4_•7H_2_O, 0.25 M NaH_2_PO_4_), 0.1 mM X-gal, and lack of both Ura and Trp.

### Transient expression in tobacco system

The coding sequences of *CsHY5*, *CsMYB4*, *CsMYB12*, *CsMYB67*, *CsC1*, *CsKTN80.4*, and *CsTTG1* were inserted into the pGreenII 62-SK vectors as effectors. The promoter fragments of flavonoid biosynthetic genes were constructed into the pGreenII 0800-LUC vectors as reporters. The recombinant pGreenII 62-SK vectors were transformed into GV3101(pSoup-p19) competent cells as effectors, while the empty pGreenII 62-SK vectors were transformed into GV3101 (pSoup-p19) as control. Equal volumes of effectors and reporters were mixed for transient transfection. The control and experimental groups were injected into the same leaf of tobacco (*N. benthamiana*) using 5–6 replicates for each group. After 48 hours (long-day white light illumination), 0.5 mM luciferin (Shanghai Macklin Biochemical Co., Ltd, China) was injected into the infiltrated places and the tobacco leaves were kept in dark for 5 minutes. Afterwards, the fluorescence signals of them were examined by SH-523 Chemiluminescence Imaging System (Shenhua Science Technology Co., Ltd, Hangzhou, China) and the fluorescence intensity was calculated using Image J software (National Institutes of Health, Germany).

### Y2H assay

To verify the interaction between CsMYB67 and CsTTG1, the coding sequence of *CsMYB67* was inserted into the pGADT7 vector, and the coding sequence of *CsTTG1* was recombined into the pGBKT7 vector. The constructed vectors (pGADT7-CsMYB67 and pGBKT7-CsTTG1) were co-transformed into the Y2HGold yeast competent cells, and the empty vectors were transformed as negative control. The yeast strains were cultivated on SD/−Leu/−Trp media (lack of Leu and Trp) for 2–3 days, and the positive colonies were selected and adjusted to the OD value of 0.1 for further screening on SD/−Leu/−Trp/-His/+X-α-gal/+AbA [containing 50 ng/mL X-α-gal and 200 ng/mL AbA and lack of Leu, Trp, and Histidine (His)] and SD/−Leu/−Trp/-His/−Ade/+X-α-gal/+AbA [containing 50 ng/mL X-α-gal and 200 ng/mL AbA, and lack of Leu, Trp, His and Adenine (Ade)] selective media plates.

### BiFC assay

The recombinant plasmids (CsMYB67-cEYFP and CsTTG1-nEYFP) were constructed by inserting the coding sequence of *CsMYB67* and the coding sequence of *CsTTG1* into the pSAT4A-cEYFP-N1 and pSAT4A-nEYFP-N1 vectors, respectively. The recombinant vectors pSAT4A-cEYFP-N1-CsMYB67 and pSAT4A-nEYFP-N1-CsTTG1 were co-transformed into *A. tumefaciens* GV3101 competent cells, while the empty pSAT4A-cEYFP-N1 vectors and pSAT4A-nEYFP-N1 vectors were co-transformed into GV3101 competent as control. The control and experimental groups were injected into the leaves of tobacco (*N. benthamiana*), using 5–6 replicates for each group. After 48–72 h infiltration, the tobacco leaves were collected and examined by a confocal laser scanning electron microscope (Zeiss LSM880).

### Gene suppression of *CsMYB67* in tea leaves

The antisense oligonucleotides (AsODNs) and corresponding sense oligonucleotides (sODNs) targeting *CsMYB67* were selected using Soligo software (Table S2, see online supplementary material). AsODNs and sODNs were synthesized by Sangon Biotech. The tea leaves were collected from tea plants cv. *Fudingdabaicha* in the middle of the day on 3 May 2023. To silence *CsMYB67* in the tea leaves, AsODNs solution (20 μM) was injected into the leaves according to the previous method [50], using the sODNs as control. After 24 h incubation, the leaves were harvested for the subsequent experiments. Three independent biological replicates were collected for quantitative real-time PCR analysis and flavonoid content analysis.

### Quantitative real-time PCR analysis

First-strand cDNA was synthesized for each of the RNA samples (1 μg). Specific primers (Table S2, see online supplementary material) were designed by NCBI Primer-BLAST based on the sequences of *Camellia sinensis* cv. ‘Fudingdabaicha’. Quantitative real-time PCR (qPCR) cycling was performed on an Applied Biosystems™ StepOnePlus™ Real-Time PCR System (Applied Biosystems™ ABI, Carlsbad, California, USA) according to the following procedure: 95°C for 2 minutes; 40 cycles at 95°C for 3 seconds; 60°C for 30 seconds, using β-actin as an internal control and three technical replicates.

### Data analysis

The data were presented as the mean value ± standard deviation. The significant difference analysis was carried out by the SPSS Statistics 25 software (IBM Inc., Chicago, IL, USA), using Tukey test.

## Supplementary Material

Web_Material_uhad231

## Data Availability

Sequencing data of this study (Accession number: CRA004002) can be found at the BIG data center (https://bigd.big.ac.cn/) under the project of No. PRJCA004626.
