# Supplementary material for: CsMYB67 participates in the flavonoid biosynthesis of summer tea leaves
Source: Hortic Res. 2023 Nov 17;11(1):uhad231. doi: 10.1093/hr/uhad231 (PMC10822840; doi:10.1093/hr/uhad231)
Supplement: Web_Material_uhad231 [file web_material_uhad231.docx]

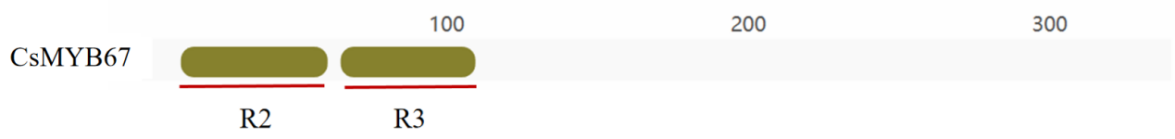


A

B


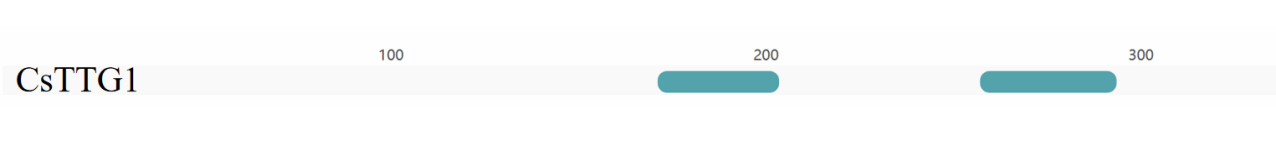


**Figure S1.** Conserved domain analysis of CsMYB67 (A) and CsTTG1 (B).


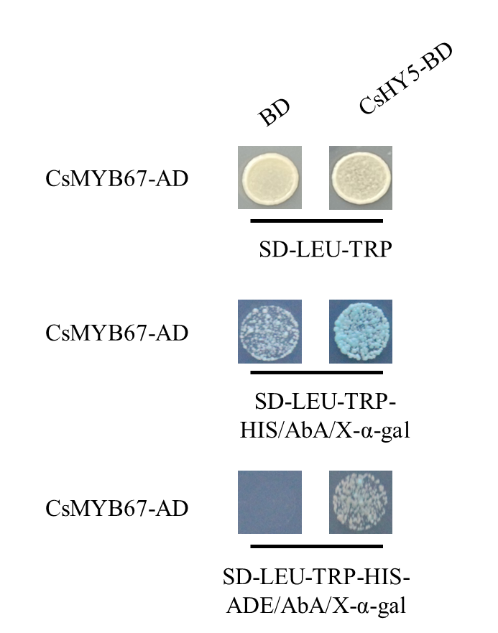
A B C


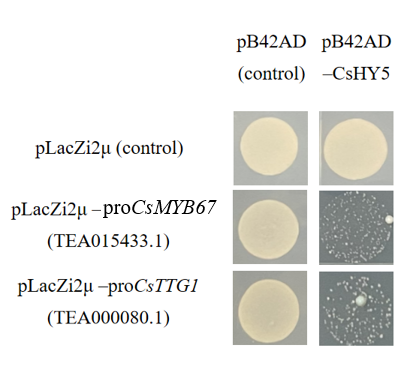


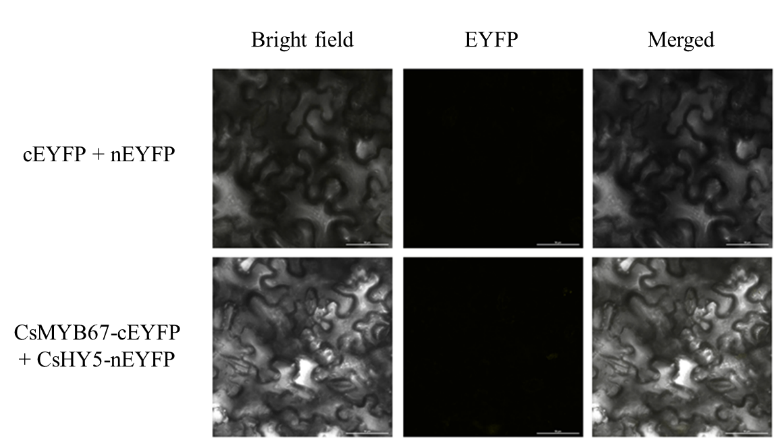


**Figure S2.** Interactions of CsHY5 with CsMYB67 or the promoter of *CsMYB67*. **A** The Y2H result of CsHY5 and CsMYB67. **B** The BiFC result of CsHY5 and CsMYB67. Scale bar=50 µm. **C** The Y1H result of CsHY5 and the promoter of *CsMYB67*.


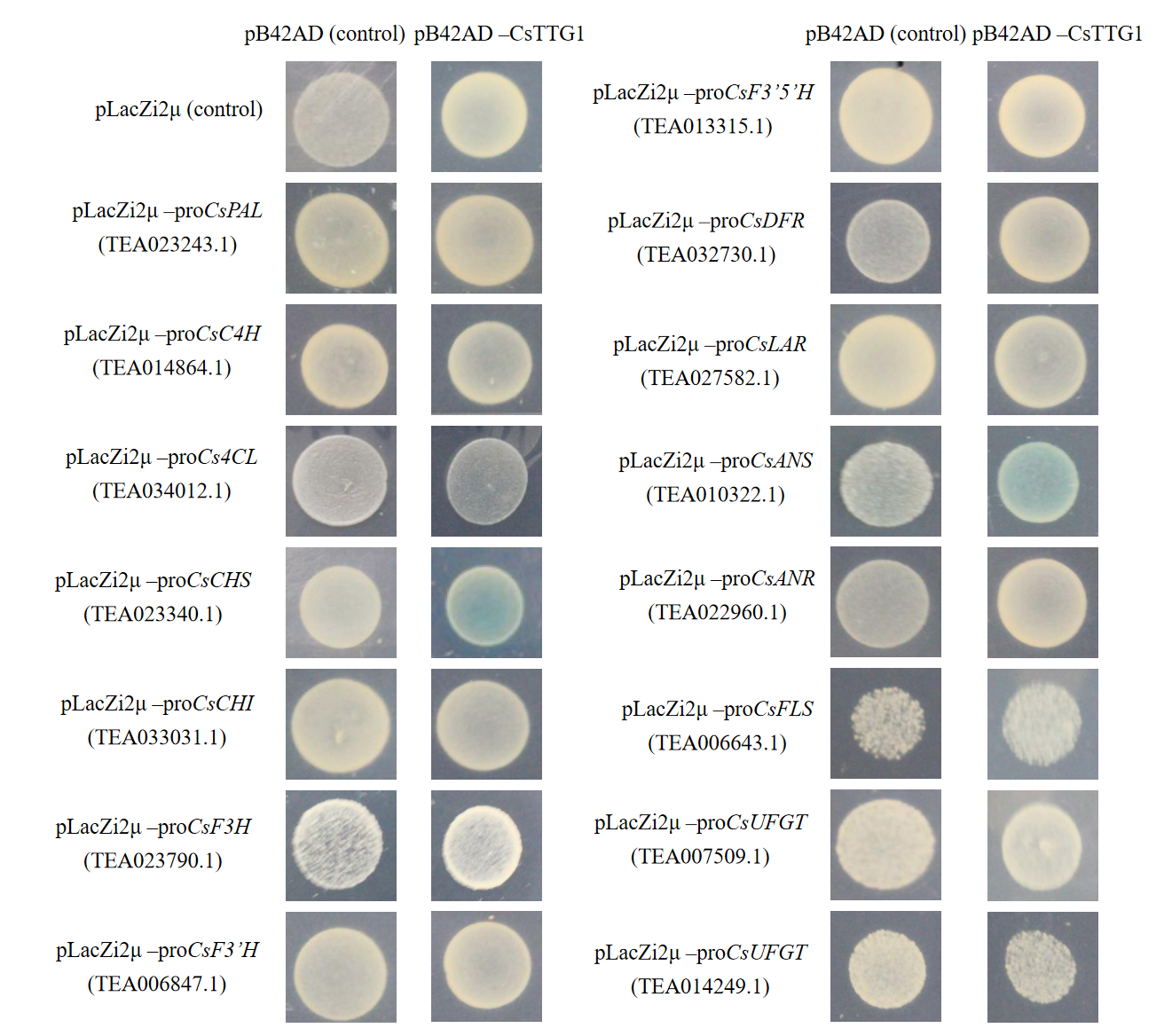


**Figure S3.** Characterization of CsTTG1 interactions with the promoters of structural genes for flavonoids biosynthesis using Y1H assay. pLacZi2μ and pB42AD were used as negative controls. SD/Gal/Raf/-Ura/-Trp/+X-gal selective media is the yeast nitrogen base containing galactose, raffinose, X-gal but lack of Ura and Trp.

**Table S1 The contents of flavonoids in the control and *CsMYB67*-silenced tea leaves**

| Compounds | | SODN | | | | ASODN |  |
| --- | --- | --- | --- | --- | --- | --- | --- |
| **Catechins (mg/g DW)** | | |  |  |  |  |  |
| GC | 2.05±0.12 | | | | 3.15±0.25** | |  |
| EGC | 68.63±3.72 | | | | 71.91±3.38 | |  |
| C | 0.94±0.06 | | | | 0.82±0.07 | |  |
| EC | 12.94±0.22 | | | | 17.11±1.53 | |  |
| EGCG | 77.66±2.35 | | | | 96.01±3.46** | |  |
| GCG | 2.09±0.16 | | | | 2.18±0.09 | |  |
| ECG | 18.57±0.42 | | | | 21.14±1.64 | |  |
| CG | 0.11±0.00 | | | | 0.12±0.00 | |  |
| TC | 182.99±3.92 | | | | 212.45±7.80** | |  |
| **Flavonol glycosides (μg/g DW)** | | |  |  |  |  |  |
| M-gal-rha-glu | 563.87±14.70 | | | | 483.48±34.39* | |  |
| M-gal | 807.06±21.81 | | | | 777.00±46.40 | |  |
| M-glu | 1571.53±124.71 | | | | 1189.28±31.85* | |  |
| Q-gal-rha-glu | 3272.13±20.34 | | | | 3810.88±105.18** | |  |
| Q-glu-rha-glu | 3155.07±157.49 | | | | 3302.32±142.74 | |  |
| Q-glu-rha-rha | 195.58±6.44 | | | | 170.21±19.89 | |  |
| Q-glu-rha | 1446.25±31.48 | | | | 1632.48±104.31 | |  |
| Q-gal | 170.65±11.78 | | | | 212.10±17.45 | |  |
| Q-glu | 132.36±7.39 | | | | 124.77±2.66 | |  |
| K-glu-rha-glu | 422.23±19.01 | | | | 549.34±29.94** | |  |
| K-gal | 44.53±2.93 | | | | 49.90±2.46 | |  |
| K-glu-rha | 18.59±2.81 | | | | 24.97±3.72 | |  |
| TFG | 11799.85±264.44 | | | | 12326.74±232.27 | |  |
| **Anthocyanins (μg/g DW)** | | |  |  |  |  |  |
| D3Ga | 4.33±0.59 | | | | 4.82±0.65 | |  |
| C3Ga | 6.38±0.30 | | | | 7.95±1.29 | |  |
| DCZGa | 5.18±1.01 | | | | 6.44±0.58 | |  |
| CCZGa | 5.51±1.70 | | | | 5.86±0.32 | |  |
| DCGa | 15.89±0.77 | | | | 24.25±2.74** | |  |
| CCGa | 11.14±1.24 | | | | 15.17±2.11* | |  |
| Total anthocyanins | 48.43±4.62 | | | | 64.48±1.99** | |  |

Three biological replicates were performed. **P* < 0.05, ***P* <0.01. DW: dry weight; EC: epicatechin; EGC: epigallocatechin; ECG: epicatechin gallate; EGCG: epigallocatechin gallate; GC: gallocatechin; C: catechin; CG: catechin gallate; GCG:gallocatechin gallate; TC: total catechins. M-gal-rha-glu: myricetin-3-*O*-glucosyl-rhamnosyl-galactoside; M-gal: myricetin-3-*O*-galactoside; M-glu: myricetin-3-*O*-glucoside; K-glu-rha-glu: kaempferol-3-*O*-glucosyl-rhamnosyl-glucoside; K-gal: kaempferol-3-*O*-galactoside; K-glu-rha: kaempferol-3-*O*-rhamnosyl-glucoside; K-glu: kaempferol-3-*O*-glucoside; Q-gal-rha-glu: quercetin-3-*O*-glucosyl-rhamnosyl-galactoside; Q-glu-rha: quercetin-3-*O*-rhamnosyl-glucoside; Q-glu-rha-rha: quercetin-3-*O*-rhamnosyl-rhamnosyl-glucoside; Q-glu-rha-glu: quercetin-3-*O*-glucosyl-rhamnosyl-glucoside; Q-gal: quercetin-3-*O*-galactoside; Q-glu: quercetin-3-*O*-glucoside; TFG: total flavonol glycosides. D3Ga: delphinidin-3-*O*-β-D-galactopyranoside; C3Ga: cyanidin-3-*O*-β-D-galatopyranoside; DCZGa: delphinidin-3-*O*-β-D-(6-(*Z*)-*p*-coumaroyl)galactopyranoside; CCZGa: cyanidin-3-*O*-β-D-(6-(*Z*)-*p*-coumaroyl)galactopyranoside; DCGa: delphinidin-3-*O*-β-D-(6-(*E*)-*p*-coumaroyl)galactopyranoside; CCGa: cyanidin-3-*O*-β-D-(6-(*E*)-*p*-coumaroyl)galactopyranoside; TA: total anthocyanins..

**Table S2 The primers of genes used for Y1H, Y2H, transient expression, gene suppression, qPCR assays**

| Assay | Vector | Gene name | Gene ID | Primer sequence (5’→3’) | |
| --- | --- | --- | --- | --- | --- |
| Y1H | pLacZi2μ | PAL | TEA023243.1 | F | attggatcggaattcAGGATATTAGAGCCCTAGTG |
|  |  |  |  | R | agcacatgcctcgagCGTCAACAGAAAAACCACAC |
|  |  | C4H | TEA014864.1 | F | attggatcggaattcCAGGACCTTGTTGAAAGGAT |
|  |  |  |  | R | agcacatgcctcgagATTGGCGTAGGTTAGAGGGA |
|  |  | 4CL | TEA034012.1 | F | attggatcggaattcGACTCATAGTAGACCTCATG |
|  |  |  |  | R | agcacatgcctcgagGCAGAAGCCACGGACAACAT |
|  |  | CHS | TEA023340.1 | F | attggatcggaattcCCTCGGCTAAGAGGTGCGAT |
|  |  |  |  | R | agcacatgcctcgagATGTCCTCGACGGTGACCAT |
|  |  | CHI | TEA033031.1 | F | attggatcggaattcCTCTAGTCAGTAATTTCCCC |
|  |  |  |  | R | agcacatgcctcgagTCCTCCACGGTGGTTGCCAT |
|  |  | F3H | TEA023790.1 | F | attggatcggaattcGTCTATACACCGACCGTAAC |
|  |  |  |  | R | agcacatgcctcgagAGCGTTGTTGTTGGCGCCAT |
|  |  | F3’H | TEA006847.1 | F | attggatcggaattcTCTAATGGGGATCGGGTTTC |
|  |  |  |  | R | agcacatgcctcgagATGAGCAGAGAGTAGAGAAC |
|  |  | F3’5’H | TEA013315.1 | F | attggatcggaattcGAAAAGGCAATGTCTTCACG |
|  |  |  |  | R | agcacatgcctcgagAAGACTGTGTCTAGGGCCAT |
|  |  | DFR | TEA032730.1 | F | attggatcggaattcTTGTAGATCAAGAAGAGCAC |
|  |  |  |  | R | agcacatgcctcgagGAAGCAACAGAGTCTTTCAT |
|  |  | LAR | TEA027582.1 | F | attggatcggaattcGCGATTATTCTCGCAATGAC |
|  |  |  |  | R | agcacatgcctcgagACACAGATTCCAACACAGTC |
|  |  | ANS | TEA010322.1 | F | attggatcggaattcAGATGGTGCATGCGCCTGGA |
|  |  |  |  | R | agcacatgcctcgagCCTGCCACAGTAGCCACCAT |
|  |  | ANR | TEA022960.1 | F | attggatcggaattcCCACTCCCCCAGGATAGTAT |
|  |  |  |  | R | agcacatgcctcgagGCTGTCGGTTGGGCTTCCAT |
|  |  | FLS | TEA006643.1 | F | attggatcggaattcGATTTCGGACCCTCAAACAT |
|  |  |  |  | R | agcacatgcctcgagTGCACTCTCTCTACCTCCAT |
|  |  | UFGT | TEA014249.1 | F | attggatcggaattcAAGCTCGATGATGTACTGTG |
|  |  |  |  | R | agcacatgcctcgagGGACGTGATGGTGATTCCAT |
|  |  | UFGT | TEA007509.1 | F | attggatcggaattcGTTGTCGGAGATGGCAATA |
|  |  |  |  | R | agcacatgcctcgagGGTTGGTTTTGTGGGATCAC |
|  |  | UFGT | TEA002089.1 | F | attggatcggaattcCCATTGGATGATCGAGATCT |
|  |  |  |  | R | agcacatgcctcgagTCTTATGAGCTGTTTGGCTA |
|  |  | UFGT | TEA010338.1 | F | attggatcggaattcCAACGAAAGCACATCGTCGT |
|  |  |  |  | R | agcacatgcctcgagTTCAATGCTCTTTGGCTCAT |
|  |  | UFGT | TEA025792.1 | F | attggatcggaattcCACAAGTACACAACGATGTG |
|  |  |  |  | R | agcacatgcctcgagGAGAAGAGGGTGCAATGGAT |
|  |  | UFGT | TEA033414.1 | F | attggatcggaattcCTTCTCTGACAATAACACGT |
|  |  |  |  | R | agcacatgcctcgagGCCATGAATGGCAACATCAC |
|  |  | UFGT | TEA025793.1 | F | attggatcggaattcTGTGAGTGCAATTTGGGACC |
|  |  |  |  | R | agcacatgcctcgagGATCTTCTCCACTCCTAGAG |
|  | pB42AD | MYB4 | TEA033191.1 | F | gcctctcccgaattcATGAGAAAACCTTGTTGTGA |
|  |  |  |  | R | ccaaagcttctcgagTCAGTTCATAGCTAAGGACA |
|  |  | MYB12 | TEA009412.1 | F | gcctctcccgaattcATGGGAAGAGCACCTTGCTG |
|  |  |  |  | R | ccaaagcttctcgagTCAAGAGAGAAGCCAAGCAA |
|  |  | MYB67 | TEA015433.1 | F | gcctctcccgaattcATGGGCCATCATTGTTGCAG |
|  |  |  |  | R | ccaaagcttctcgagTTAACAATCCCATGCAAGTT |
|  |  | C1 | TEA004608.1 | F | gcctctcccgaattcATGGGGAGGAGTCCATGCTG |
|  |  |  |  | R | ccaaagcttctcgagTCATGGCCAGTCCTCAGAAT |
|  |  | KTN80.4 | TEA033903.1 | F | gcctctcccgaattcATGTTACGTGGCGACTTACA |
|  |  |  |  | R | ccaaagcttctcgagTCATGAGTTTTGAAGAACTA |
|  |  | TTG1 | TEA000080.1 | F | gcctctcccgaattcATGGAGAATTCGAGCCAAGA |
|  |  |  |  | R | ccaaagcttctcgagTCAAACTTTCAGAAGCTGCA |
| Y2H | pGADT7 | MYB4 | TEA033191.1 | F | gccagtgaattccacccgggtATGAGAAAACCTTGTTGTGA |
|  |  |  |  | R | gagctcgatggatccTCAGTTCATAGCTAAGGACA |
|  |  | MYB12 | TEA009412.1 | F | gccagtgaattccacccgggtATGGGAAGAGCACCTTGCTG |
|  |  |  |  | R | gagctcgatggatccTCAAGAGAGAAGCCAAGCAA |
|  |  | MYB67 | TEA015433.1 | F | gccagtgaattccacccgggtATGGGCCATCATTGTTGCAG |
|  |  |  |  | R | gagctcgatggatccTTAACAATCCCATGCAAGTT |
|  |  | C1 | TEA004608.1 | F | gccagtgaattccacccgggtATGGGGAGGAGTCCATGCTG |
|  |  |  |  | R | gagctcgatggatccTCATGGCCAGTCCTCAGAAT |
|  |  | KTN80.4 | TEA033903.1 | F | gccagtgaattccacccgggtATGTTACGTGGCGACTTACA |
|  |  |  |  | R | gagctcgatggatccTCATGAGTTTTGAAGAACTA |
|  |  | TTG1 | TEA000080.1 | F | gccagtgaattccacccgggtATGGAGAATTCGAGCCAAGA |
|  |  |  |  | R | gagctcgatggatccTCAAACTTTCAGAAGCTGCA |
|  | pGBKT7 | HY5 | TEA012075.1 | F | atggaggccgaattcATGCAAGAACAAGCAACGAG |
|  |  |  |  | R | caggtcgacggatccCTACTTCCTACCCTCCTGCA |
|  |  | TTG1 | TEA000080.1 | F | atggaggccgaattcATGGAGAATTCGAGCCAAGA |
|  |  |  |  | R | caggtcgacggatccTCAAACTTTCAGAAGCTGCA |
| Transient expression | pGreenⅡ 0800-LUC | PAL | TEA023243.1 | F | ggcgaattgggtaccAGGATATTAGAGCCCTAGTG |
|  |  |  |  | R | accgtcgacctcgagCGTCAACAGAAAAACCACAC |
|  |  | C4H | TEA014864.1 | F | ggcgaattgggtaccCAGGACCTTGTTGAAAGGAT |
|  |  |  |  | R | accgtcgacctcgagATTGGCGTAGGTTAGAGGGA |
|  |  | 4CL | TEA034012.1 | F | ggcgaattgggtaccGACTCATAGTAGACCTCATG |
|  |  |  |  | R | accgtcgacctcgagGCAGAAGCCACGGACAACAT |
|  |  | CHS | TEA023340.1 | F | ggcgaattgggtaccCCTCGGCTAAGAGGTGCGAT |
|  |  |  |  | R | accgtcgacctcgagATGTCCTCGACGGTGACCAT |
|  |  | CHI | TEA033031.1 | F | ggcgaattgggtaccCTCTAGTCAGTAATTTCCCC |
|  |  |  |  | R | accgtcgacctcgagTCCTCCACGGTGGTTGCCAT |
|  |  | F3H | TEA023790.1 | F | ggcgaattgggtaccGTCTATACACCGACCGTAAC |
|  |  |  |  | R | accgtcgacctcgagAGCGTTGTTGTTGGCGCCAT |
|  |  | F3’H | TEA006847.1 | F | ggcgaattgggtaccTCTAATGGGGATCGGGTTTC |
|  |  |  |  | R | accgtcgacctcgagATGAGCAGAGAGTAGAGAAC |
|  |  | F3’5’H | TEA013315.1 | F | ggcgaattgggtaccGAAAAGGCAATGTCTTCACG |
|  |  |  |  | R | accgtcgacctcgagAAGACTGTGTCTAGGGCCAT |
|  |  | DFR | TEA032730.1 | F | ggcgaattgggtaccTTGTAGATCAAGAAGAGCAC |
|  |  |  |  | R | accgtcgacctcgagGAAGCAACAGAGTCTTTCAT |
|  |  | LAR | TEA027582.1 | F | ggcgaattgggtaccGCGATTATTCTCGCAATGAC |
|  |  |  |  | R | accgtcgacctcgagACACAGATTCCAACACAGTC |
|  |  | ANS | TEA010322.1 | F | ggcgaattgggtaccAGATGGTGCATGCGCCTGGA |
|  |  |  |  | R | accgtcgacctcgagCCTGCCACAGTAGCCACCAT |
|  |  | ANR | TEA022960.1 | F | ggcgaattgggtaccCCACTCCCCCAGGATAGTAT |
|  |  |  |  | R | accgtcgacctcgagGCTGTCGGTTGGGCTTCCAT |
|  |  | FLS | TEA006643.1 | F | ggcgaattgggtaccGATTTCGGACCCTCAAACAT |
|  |  |  |  | R | accgtcgacctcgagTGCACTCTCTCTACCTCCAT |
|  |  | UFGT | TEA014249.1 | F | ggcgaattgggtaccAAGCTCGATGATGTACTGTG |
|  |  |  |  | R | accgtcgacctcgagGGACGTGATGGTGATTCCAT |
|  |  | UFGT | TEA007509.1 | F | ggcgaattgggtaccGTTGTCGGAGATGGCAATA |
|  |  |  |  | R | accgtcgacctcgagGGTTGGTTTTGTGGGATCAC |
|  |  | UFGT | TEA002089.1 | F | ggcgaattgggtaccCCATTGGATGATCGAGATCT |
|  |  |  |  | R | accgtcgacctcgagTCTTATGAGCTGTTTGGCTA |
|  |  | UFGT | TEA010338.1 | F | ggcgaattgggtaccCAACGAAAGCACATCGTCGT |
|  |  |  |  | R | accgtcgacctcgagTTCAATGCTCTTTGGCTCAT |
|  |  | UFGT | TEA025792.1 | F | ggcgaattgggtaccCACAAGTACACAACGATGTG |
|  |  |  |  | R | accgtcgacctcgagGAGAAGAGGGTGCAATGGAT |
|  |  | UFGT | TEA033414.1 | F | ggcgaattgggtaccCTTCTCTGACAATAACACGT |
|  |  |  |  | R | accgtcgacctcgagGCCATGAATGGCAACATCAC |
|  |  | UFGT | TEA025793.1 | F | ggcgaattgggtaccTGTGAGTGCAATTTGGGACC |
|  |  |  |  | R | accgtcgacctcgagGATCTTCTCCACTCCTAGAG |
|  | pGreenⅡ 62-SK | MYB4 | TEA033191.1 | F | gggctgcaggaattcATGAGAAAACCTTGTTGTGA |
|  |  |  |  | R | gggccccccctcgagTCAGTTCATAGCTAAGGACA |
|  |  | MYB12 | TEA009412.1 | F | gggctgcaggaattcATGGGAAGAGCACCTTGCTG |
|  |  |  |  | R | gggccccccctcgagTCAAGAGAGAAGCCAAGCAA |
|  |  | MYB67 | TEA015433.1 | F | gggctgcaggaattcATGGGCCATCATTGTTGCAG |
|  |  |  |  | R | gggccccccctcgagTTAACAATCCCATGCAAGTT |
|  |  | C1 | TEA004608.1 | F | gggctgcaggaattcATGGGGAGGAGTCCATGCTG |
|  |  |  |  | R | gggccccccctcgagTCATGGCCAGTCCTCAGAAT |
|  |  | KTN80.4 | TEA033903.1 | F | gggctgcaggaattcATGTTACGTGGCGACTTACA |
|  |  |  |  | R | gggccccccctcgagTCATGAGTTTTGAAGAACTA |
|  |  | TTG1 | TEA000080.1 | F | gggctgcaggaattcATGGAGAATTCGAGCCAAGA |
|  |  |  |  | R | gggccccccctcgagTCAAACTTTCAGAAGCTGCA |
|  |  | HY5 | TEA012075.1 | F | gggctgcaggaattcATGCAAGAACAAGCAACGAG |
|  |  |  |  | R | gggccccccctcgagCTACTTCCTACCCTCCTGCA |
| Gene suppression | | sODN-MYB67-1 | | CTAAACTAGCAGGCTTGCAA | |
|  |  | sODN-MYB67-2 | | AGATAGCCAAACACCTCCCT | |
|  |  | sODN-MYB67-3 | | AACACCTCCCTGGTAGAACT | |
|  |  | sODN-MYB67-4 | | CCATTACTACTGCTTCTACC | |
|  |  | sODN-MYB67-5 | | GGAACTCATGCATCAAGAAA | |
|  |  | sODN-MYB67-6 | | TCCCTGGTAGAACTGACAAT | |
|  |  | AsODN-MYB67-1 | | TTGCAAGCCTGCTAGTTTAG | |
|  |  | AsODN-MYB67-2 | | AGGGAGGTGTTTGGCTATCT | |
|  |  | AsODN-MYB67-3 | | AGTTCTACCAGGGAGGTGTT | |
|  |  | AsODN-MYB67-4 | | GGTAGAAGCAGTAGTAATGG | |
|  |  | AsODN-MYB67-5 | | TTTCTTGATGCATGAGTTCC | |
|  |  | AsODN-MYB67-6 | | ATTGTCAGTTCTACCAGGGA | |
| qPCR | | β-actin | TEA019484.1 | F | CTTCCTCATGCTATCCTCCGTCTT |
|  |  |  |  | R | ATTTCCCGTTCAGCAGTGGTG |
|  |  | MYB67 | TEA015433.1 | F | GGTCTCCTGAAGAAGATGAGAAG |
|  |  |  |  | R | CTTTGCAAGCCTGCTAGTTTAG |
|  |  | FLS | TEA006643.1 | F | CCCTCGGAGTTGAACCTCAC |
|  |  |  |  | R | ACGACAAACACAGCCCAAGA |
|  |  | UFGT | TEA007509.1 | F | GAACTCCTTCGAAGAGCTAGAG |
|  |  |  |  | R | CTGATGAGGCTGGTGATGATAG |
|  |  | ANS | TEA010322.1 | F | AACAAGCGAGTACGCAAAGC |
|  |  |  |  | R | TGAAGCTCTTCCATGCCTCC |
